# The quality of life of people with ASD through physical activity and sports

**DOI:** 10.1016/j.heliyon.2022.e09193

**Published:** 2022-03-25

**Authors:** José Luis Cuesta-Gómez, Raquel De la Fuente-Anuncibay R, Ruth Vidriales-Fernández, Maria Teresa Ortega-Camarero

**Affiliations:** a Department of Education, University of Burgos, 09001, Burgos, Spain; b Confederación Autismo España, Madrid, Spain; c Department of Private Right, University of Burgos, 09001, Burgos, Spain

**Keywords:** Physical activity, Sport, Quality of life, Autism spectrum disorder

## Abstract

Among the factors that influence the quality of life of people with Autism Spectrum Disorder (ASD), physical activity and sport are key dimensions of physical well-being. Few studies take into account the perspectives of people with ASD in order to understand the extent of physical well-being and their subjective perception of it. The development of a system of quality of life indicators related to physical activity and sport for people with ASD and their analysis is the aim of this study, providing guidelines for improvement. A study was carried out with a sample of 276 people, professionals (n = 143), family members (n = 73) and people with ASD (n = 60). The people with ASD in the sample belonged to three age categories: children aged 8–12 years, adolescents aged 12–18 years and adults over 18 years. The research team used a qualitative methodology in the collection of information. They adapted three questionnaires, with validated quality of life scales, to guide the interviews and also applied them in the design of the focus group protocols. Subsequently, the research team analysed the information collected in the focus groups with families and professionals using a DELPHI method. A system for coding the responses and qualitative analysis of the responses was also prepared for the analysis of the data by the research team. Finally, the information obtained was compared with a group of representative experts. The results concluded with the elaboration of a system of quality of life indicators related to the dimension of physical well-being, as well as guidelines and proposals that bring together the perspectives of people with ASD in relation to the practice of sport and physical activity. There is a need to increase the practice of sport among people with ASD in order to promote their health, social participation and personal satisfaction.

It is concluded that it is not possible to obtain a broad picture of the quality of life of people with ASD and their families due to lack of information. However, the method and the results obtained represent a first approach at national level to increase knowledge about the quality of life of people with ASD.

## 1 Introduction

The conceptual development of the Quality-of-Life model and its application have facilitated change and the expansion of the assistance that people with ASD receive. In that context, quality of life is understood to be “A desired state of personal wellbeing composed of various central dimensions that are influenced by personal and environmental factors. These central dimensions are equal for everybody, but can vary individually with regard to the importance and the value attributed to them” [1].

The Convention on the Rights of persons with disabilities [2], and the Quality of Life model proposed by Schalock and Verdugo [3], observes a significant relationship between quality of life and physical wellbeing, interpersonal relations, and social inclusion. In this sense, various studies have pointed out that people, both with and without ASD, who practice sport and engage in physical activity obtain cognitive, psychological and social benefits [4, 5, 6, 7]. Some authors have pointed out that sport and physical activity in people with disability improve their social skills, as well as levels of trust and self-esteem [8, 9].

Although it is clear that practicing sport improves health, participation and quality of life, as proposed by Chun et al. [10], Aniszewski et al. [11], and Goñi et al. [12], the current figures for people who have sedentary lifestyles is very high, and the situation of people with disability is alarming in this context. According to recent studies, they have higher sedentary lifestyles than people without disabilities do [13, 14].

With respect to the group of people with autism spectrum disorder (ASD), we find coincident results. They obtain better outcomes in cognitive, psychological, and behavioral aspects, as well as in social and motor functioning, when they have a regular practice of physical activities and sports [15, 16, 17]. In that sense, authors like Zhao et al. [18], and Webster [19], points out the relevance of physical wellbeing and health education in the case of people with ASD.

Studies indicate that children with ASD have an inclination to maintain a sedentary attitude that, together with low levels of physical activity, is associated with negative short- and long-term health consequences. In this regard, some systematic reviews of research [20], associating the prevalence of sedentary behaviours and low levels of physical activity in children with ASD, estimated a physical activity of 86 min per day; and the prevalence of sedentary behaviour (271 min/day in front of the screen and 479 min/day) in total sedentary behaviour. Age was inversely associated with physical activity, with decreasing levels of physical activity with increasing age and school level. Regarding the relationship between sex and physical activity, some authors point to an inconsistency in studies among children with ASD [21, 22, 23]. Among typically developing populations, boys are more active than girls, but as children get older, the influence of gender is less among children with ASD. However, given the paucity of studies, more research is needed to understand more fully whether or not boys with ASD are more active than girls with ASD.

Compared to the general population, some studies indicate that few children participate in at least 60 min per day of the recommended moderate to vigorous intensity physical activity [24]. Compared to children with ASD, the risk of inactivity is even higher, as in the case of children (0–18 years) diagnosed with an Autistic Spectrum Disorder [20].

In this line, the research carried out by García-Gómez et al. [25], insists on the need to carry out practices that promote physical activity for people with ASD, as these have a clear effect on the health, sleep and quality of life of people with ASD. Some authors point out that the improvement in sleep patterns in people with ASD is accompanied by a reduction in behavioural symptoms, and improvements in cognitive-executive dimensions and motor functioning [26, 27, 28, 29].

For their part, Bernate et al. [30], point out in their study that the benefits of physical activity include emotional stability, increased euphoria and self-esteem, a decrease in self-harm, tension and stress, etc. In other words, physical activity is shown to have beneficial physical and psychological effects. Their research concludes that emotional regulation and mood regulation through physical activity and social sport promote autonomy, interaction and inclusion of people with ASD in the context of interaction [31].

The results of the meta-analysis of Healy et al. [32], on the effects of physical activity on young people with ASD, considering the results of 29 studies [n = 1009], enlighten the positive effects on manipulative and motor skills, physical aptitude, social functioning, muscular development and strength among others. Likewise, some authors pointed to the need to consider physical activity as the essential principle that guarantees quality of life when people with ASD face the aging processes [33].

In addition, a recent review involving a total of 4,347 published papers yielded hardly significant benefits in communication skills (p = .139) and a significant improvement for general social functioning (p = .001) in children with ASD, related to the regular practice of sport and physical activity in small groups. However, despite the recognized limitations, the importance of physical activity in groups for children with ASD was underlined [34].

Some authors highlighted the existence of certain barriers to increased physical activity. This is due to low motivation, poor motor functioning, self-regulation and social difficulties, as well as struggles with planning and generalisation of skills in different contexts [35, 36], among other factors.

Most studies published in the last ten years have attempted to explore what factors positively influence both personal outcomes and prognosis for people with ASD [37].

These studies consider those results like positive indicators of “personal success”. However, they do not take into account how those results improve or enrich the person's quality of life. Quality of life is a concept that must require a multidimensional perspective, including many other aspects of the person's life, such as physical wellbeing, health, and leisure, and the subjective satisfaction with all of them. Traditionally, most of the studies on quality of life and ASD do not incorporate this view, and exclude the subjective perspective in the consideration of the person's achievements [38, 39].

Based on the above propositions, we set out the principal aim of the study: getting the perspectives and opinions of people with ASD and their families regarding their quality of life in the physical wellbeing dimension in order to develop practical guidelines and indicators to promote it.

## 2 Materials and methods

The research team designed a qualitative approach, based on the quality-of-life model proposed by Schalock and Verdugo [3]. This model defines individual quality of life as a complex and multidimensional phenomenon, not reduced to health-related wellness. It reflects the wellbeing desired by the person in relation to eight common domains: psychical, emotional and material wellbeing; personal development; social inclusion; interpersonal relationships; self-determination and rights. Each domain include subjective and objective indicators, and is influenced by contextual and personal factors, that interact between them and with the social and cultural environment.

### 2.1 Instruments

In order to explore the experiences and perceptions among people with ASD, their families and the professionals about the previous domains of quality of life, the research team designed and applied several qualitative techniques (semi-structured individual interviews and focus groups, mainly). To facilitate the design of the interviews protocols, the research team review the following instruments:

• Childhood Quality of Life Questionnaire (CVI-CVIP) [40].
  Global reliability CVI PSR = 0.88 (88%).
  Global reliability CVIP PSR = 0.83 (83%).
• *Quality of Life Evaluation Questionnaire for Adolescent Students* (CCVA) [41].
  Reliability α = 0.84 (84%).
• Scale INICO-FEAPS: Comprehensive Assessment of the Quality of Life of People with Intellectual or Developmental Disabilities [42].
  Reliability of "other people's report" α = 0.937 (93′7%).
  Reliability of "Self-Reporting” α = 0.893 (89′3%).

To test the reliability and validity of the scales and subscales, Cronbach's alpha consistency coefficient (α) and the Person Separation Reliability statistic are used (PSR).

All the protocols were designed to obtain information on the perception of people with ASD, relatives and professionals on the Quality of Life of people with ASD and to establish recommendations to promote it.

After that, four experts [not related to the professional network] reviewed the protocols in order to assure the pertinence and comprehension of the questions and explored domains.

All the participants were recruited through a network of autism organizations led by Autism Spain and the Burgos University. 23 professionals, with broad experience in supporting people with ASD and their relatives, took part as experts on the network.

As we can see in Table 1, the sample of interviewed participants comprised different groups: relatives of the people with ASD (n = 73); people with ASD (n = 60); and professionals (n = 143) from different organizations and areas of Spain. The inclusion criteria for the ASD group were: an age of over 8 years old, presenting a confirmed diagnosis of Asperger Syndrome (AS), autism without associated intellectual disability -Total Intellectual Quotient TIQ-assessed through standardized tests of over 70- or Generalized Developmental Disorders-non-specific without associated intellectual disability, on the basis of the criteria established in DSM-V-TR [43]. All the participants were evaluated with the *Autism Diagnostic Observation Schedule (ADOS-G)*, proposed by Lord et al. [44], exceeding the cut-off scores established for ASD. All the participants received support through the organizations linked to Autism Spain.

**Table 1:** Number of persons participating in the sample classified by category.

Family members of people with ASD		n = 73
People with ASD	Children 8–12 years old	n = 14
	Adolescents 12–18 years old	n = 22
	Adults +18 years old	n = 24
Professionals		n = 143
Total		N = 276

Regarding the parents and relatives participating in the study; their age ranged between 32 and 56 years, M = 44.23 years and SD = 6.67. With regards to their level of education, 60% had higher education, 30% had intermediate education and 10% had elementary education. The level of occupation is high, 90% of the parents have a stable job, while 10% of the parents have unstable employment.

The inclusion criteria for the participation of the organizations were to provide support services to people with ASD and to their relatives at different developmental stages -at least two.

Informed consent was obtained from study participants. All participants signed documents regarding informed consent to participate, protection of personal data and confidentiality. The research was approved by the Bioethics Committee of the University of Burgos "IR 15/2018"., guaranteing anonymity.

### 2.2 Procedure

Table 2 below shows schematically the procedure carried out in the research. This is followed by an explanation of each of the phases.

**Table 2:** Phases that make up the research.

**Phase 1. Documentary and bibliographic analysis.**				
Following a literature review, participants and data collection techniques were selected.				
**Phase 2. Application of quality of life scales to people with ASD and development of focus groups. N= 276**				
Quality of life questionnaires (QoL) according to the age of those assessed. - Duration of application: 1h (approx.).		Children 8–12 years old. n = 14		[40].
		Adolescents 12–18 years old. n = 22		[41].
		Adults +18 years old. n = 24		[42].
Focus/interest group development. N = 216 - Duration: 2h (approx.). - Format: semi-structured.		Professionals. n = 143		They come from different territorial locations, with different backgrounds and professional profiles.
		Families. n = 73		Characteristics: - At least 1 at school age. - At least 1 between 16-25 years old. - At least 1 over 25 years old. - At least 2 with ASD + ID. - At least 2 with ASD without ID. - At least 1 female with ASD.
**Phase 3. Drawing up a table of indicators with experts. N = 15**				
Categorisation and analysis/coding of information. n = 3			Group of researchers with extensive experience in working with people with ASD.	
Proposed objectives.				
Validation of objectives.				
Generation of a table of indicators of physical well-being to measure the quality of life of people with ASD in this dimension.				
Submission of the proposed objectives, the table of indicators and evidence generated to expert judgement using the Delphi technique. n = 12			Four consultations.	
			Criteria for the selection of experts: - Experience in ASD of more than 5 years. - Different backgrounds. - Representation of different organisations. - Different professional profiles. - Experience working with children, adolescents and adults.	
Descriptive characteristics of the expert group.	Gender.		Men: 4 Women: 8	
	Years of experience.		More than 3 years of experience.	
	Provenance.		Different territorial representation.	
	Qualification.		Professionals in the fields of education and health (psychology).	

The first phase in the procedure was to obtain the information about the subjective perception of the participants about the quality of life of people with ASD, though the personal interviews and focus groups in the different organizations. The interviews took part in the natural context of the person with ASD. An experienced professional carried out the interviews. In all cases, the professional was also familiar to the participant and had supported him or her for at least 12 months.

The research team carried out several semi-structured interviews and focus groups with relatives of people with autism spectrum disorder in order to obtain information about their experiences in providing support to the families of people with this disorder. The focus groups followed a similar protocol as the individual interviews of the people with ASD did, in order to assure that everybody shared the main conversation topics. The information from the professionals was obtained individually.

After that, the research team systematized and analyzed all the information through a DELPHI technique, contrasting it with the experts in the network.

The DELPHI technique is a qualitative sociological consensus method, belonging to the in-depth interview group, allowing the collection of subjective information based on the criteria and opinions of a group of experts. It is aimed at understanding informants' perspectives on a research topic, without the need to generate direct interaction between its components within a physical space [45, 46, 47, 48, 49, 50].

The last step in the procedure was to develop the agreed strategies and recommendations arising from experience and professional practice oriented to promote the physical activity and sport practice as key aspects of the physical wellbeing of people with ASD.

The research team carried out a qualitative coding and analysis of the information provided in the interviews and discussion groups, considering the representation of the experiences and perspectives of the participants (people with autism spectrum disorder, family members and professionals) in relation to the quality of life.

The research team developed the analysis process in four phases.

Phase 1. Literal transcription of interviews and discussion groups, and assignment of identification codes.

Phase 2. Coding of statements and expressions, and thematic classification of verbalizations based on the quality of life dimensions [3].

Phase 3. Content analysis, following the guidelines of the discourse analysis and the identification of discursive positions (agreements and disagreements) between the different interest groups: people with autism spectrum disorder, relatives and professionals.

The analysis of the information showed the redundancy of the representations expressed by the participants, fulfilling the discourse saturation criteria.

Phase 4. Definition of areas (first order categories) and indicators (second order categories) related to the quality of life dimensions.

This group was integrated by eight women and four men, and they participated and validated the entire content analysis process and the achieved results. All the experts had more than 3 years of proffesional experience, ranging between 3 and 23 years of expertise. The average years of expertise within the group was of 12.25 years.

Using a DELPHI technique, this group also validated the first and second order categories. They also clarified and decided how to proceed (to reclasiffied or to delete) with the responses that were difficult to classify in a given category.

## 3 Results

The qualitative analysis identified the key elements related to the dimensions that Schalock & Verdugo [3], defines in their quality of life model in the specific case of people with autism spectrum disorder.

In this paper, we only present the results related to the *physical wellbeing dimension and specifically those regarding to the impact of physical activity and sport in the physical wellbeing.*

The main conclusions achieved in the qualitative data analysis of the *interviews* and *Discussion Groups* are:

- Families and professionals agree than people on the autism spectrum should practice a regular physical activity or sport. People with autism spectrum disorder also express their interest in practicing a wide range of physical activities, both informal (walking….) and organized sports (playing football, swimming…).
  o “In my opinion, it's important to enjoy with the physical activity that you choose, and it's essential to have time to practice it”. Man with autism spectrum disorder, 33 years old.
  o “*I enjoy going to the swimming pool and cycling*”. Man with autism, spectrum disorder, 28 years old.
  o “*It´s important to be healthy. I try to keep a good health by practicing sport several times a week and eating well*”. Man with autism spectrum disorder, 29 years old.
- Families and professionals perceive that practicing a physical activity reduces stress in the case of some people in the autism spectrum. Practicing regular exercise or sports also contribute to maintain psychomotor skills, and to improve socialization and personal satisfaction.
  o “*He is happy when he goes to the swimming pool or goes for a walk, especially in the countryside*”. Mother of an adult man with autism spectrum disorder.
  o “*She needs to practice an intense and regular physical activity. It´s important to her wellbeing and her behavior, and also for ourselves too*”. Father of an adult woman with autism spectrum disorder.
  o *"He has to do moderate daily physical activity, including walking, swimming, or biking. These activities help him relax and benefit him a lot".* Mother of an adolescent with autism spectrum disorder.
- People with autism spectrum disorder should combine the practice of a daily physical activity (such as walking) with other exercise or leisure alternatives (games and sports). In both cases (physical activity and sport practice), the activity should be adapted to age, physical condition and individual preferences.
- These sports should be adapted whenever necessary, including supporting materials (visual clues, alternative or augmentative communication systems, clear schedules and instructions…), and specialized professionals that facilitate the participation of people on the autism spectrum.
  o “*Our children need a specialized support in the sport practice. They need professionals to support them, and these professionals must know about autism, and they have to know how to help and motivate our children too*”. Mother of an adolescent with autism spectrum disorder.
- People with autism spectrum disorder should enjoy the same opportunities and alternatives than any other citizen in the access and use of sport facilities. These facilities should provide adjustments and adaptations on the environment (cognitive and physical accessibility) when necessary, in order to guarantee its easy comprehension and easy use, as well as the safety of people with autism spectrum disorder.
  o *“Our children must be able to access the sports centers with the same opportunities as other people. And they should have the supports they need to understand the environment and to know how to perform the exercises*". Mother of an adult man with autism spectrum disorder.
- The sport or leisure facilities and their staff should provide the individualized support that people on the autism spectrum may need to participate and enjoy the activities, without discrimination and in the same conditions than other users.
  o “*If it is possible, I think it´s important to encourage the person to enjoy in sports competition. It gives him or her the opportunity to be part of “normalized*” contexts”. Professional.
  o “*The sport practice offers unique opportunities for the social inclusion*”. Professional.
- The cost of the sport activities has to be affordable for people with autism spectrum disorder and their families, and it cannot suppose an additional barrier to the activity practice.
  o “*We have to expend a lot of money because the autism. Some sport activities are expensive, and this is a barrier for us*”. Father of an adolescent with autism spectrum disorder.
- In some cases, people with autism spectrum disorder may need a specialized care to ensure their physical wellbeing. In these cases, they should have access to physiotherapy care or physical rehabilitation. The providers of this type of health care should ensure the adjustments and supports that people with autism could need to benefit from it.
  o “*Sport practice must be adapted to the individual need and personal circumstances. Some people with autism maintain negative habits in the care of their health along their lives, so it is important to be careful if we support them in the sport practice. They have higher risks to be hurt or to develop a sport injury*”. Professional.
  o “*Some people with autism may need specific care regarding their physical wellbeing, especially physiotherapy or other types of care to help with non-healthy habits, like bad corporal postures*”. Professional.

The research team organized this information, as well as and the first-order categories (areas) and the second-order categories (factors) in a structured model with three components: objectives, indicators, and evidences.

There is an example in Table 3 that shows the correspondence between the categories and elements described above regarding the physical wellbeing and the practice of physical activity and sports (area-objective in the area-indicators of achievement- and evidence to contrast the achievement).

**Table 3:** Quality of Life Indicators in the dimension of physical wellbeing (physical exercise and sport).

Area- physical activity and sport practice		
Objective	Indicator	Examples/evidences
1. To increase the practice of a varied range of healthy and inclusive physical activities	1.1. The weekly schedule of exercise and physical activity takes into account the age, physical condition and individual preferences of the person	- The person can choose between various physical or sport activities, according to his/her preferences. - There are recommendations (type of sport activity, frequency, intensity, or others), related to the person age or physical condition.
	1.2. The physical activities or sport practice take place in community settings.	- The person attends community sport facilities in order to enjoy their available activities. - There are individualized supports systems in the community settings that facilitate the person participation. - The person has the opportunity to interact with other people through the practice of physical activities. - The person is encourage to participate in competitions or sport events, if he/she is interested.
2. To improve the person's physical condition.	2.1. The person attends physiotherapeutic services if needed.	- The person can visit a physiotherapist with experience and knowledge related to autism spectrum disorder. - The physiotherapist follows the physical condition or discomfort regularly. - The physiotherapist trains the person (and/or his/her relatives) in order to promote a healthy corporal posture and to reduce discomfort or pain.
	2.2. The person improves his/her physical wellbeing	- The person loses weight (if needed) - The values in blood tests improve or get better (if needed)

After completing the Quality of Life system of indicators, the research team and the group of experts developed a guideline to promote and increase the physical activity and the sport practice in the case of people with autism spectrum disorders. The objective of these guidelines is to facilitate the practical application of the indicators, and monitoring its impact on the quality of life of people with autism spectrum disorder.

Table 4 summarize the main recommendations and guidelines regarding physical activity and sport practice.

**Table 4:** Guidelines to improve physical wellbeing.

Guidelines to improve physical wellbeing and sports practice among peopleo with autism sepctrum disprder
- The physical activities or exercises must be functional and meaningful for the person with autism spectrum disorder. The person must understand the activity rules, must know how to perform it and how long is going to take. Understanding the activity is essential for maintaining the person's motivation. - The activity goals should be clear and explicit. The person role during the activity should be clear too, so encourage him or her to enroll in predictable activities with plain instructions. - The communication should be clear and unambiguous. The instructions should be brief and precise, avoiding the non-literally language (metaphors, irony…). Other supports, like alternative or augmentative communication systems, can help the person to understand the instructions and follow them. - Sometimes the person can feel anxious or concern during the physical activity. You can support her or him in these situations, postponing or restructuring the activity in order to give the person a chance to rest or relax. - The environment comprehension and its cognitive accessibility are essentials for the person with autism spectrum disorder. The comprehension of physical spaces, schedules, symbols or written information will enhance the independence and autonomy in the sport practice. - Use contextually specific and appealing materials in accordance to the age of the person with autism spectrum disorder. Do not infantilize her or him. - One of the key elements in the sport practice is to achieve a routine. This routine will help the person with autism spectrum disorder to feel comfortable with the regular practice and will increase his or her feeling of security and predictability. - Provide a guide to develop the activity, beyond the physical development or support. Help the person to improve her or his performance in the sport practice and to achieve independence and experience in the field. - We should understand the contingency of behaviors, so make sure you promote a “free of mistakes” learning approach. It will be easier for the person to learn in this way. - Practicing a sport or physical activity can be very hard for some people, especially at the beginning. In order to promote engagement and motivation, we should try hard to support positive feelings and to avoid failure experiences for the person with autism spectrum disorder. Empathy plays an essential role in this matter.

Authors' own work acknowledging Martínez et al. [51] and the results of the investigation.

## 4 Discussion

This study has captured the subjective perspective of people with ASD, their families and specialized professionals regarding the physical wellbeing, and its relation to the sport and physical activity practice.

Physical wellbeing is a key dimension in the quality of life of every person. Studies on healthy lifestyle and sports activities during the whole life associate the physical activity with the promotion of physical and mental health [52, 53].

This is shown by the results of the study by Lang et al. [54], which suggests that the level of physical activity is also related to sleep quality and sleep patterns in children and adolescents with ASD.

These conclusions are coincident in the case of people with ASD. The lack of physical activity and a sedentary lifestyle increase the risk of obesity, cardiovascular issues, respiratory difficulties, and other chronic conditions that compromise health, wellbeing and life expectancy [55, 56].

In accordance with our results that point to the need to increase actions focused on improving the physical well-being of people with ASD, numerous studies relate deficits in the development of motor skills, physical aptitudes and possible pathologies such as obesity that interfere with their quality of life [57]. In the same vein, Srinivasan et al. [58], point out that children with ASD are more prone to sedentary activity, this lack of physical activity in the long term can lead to the development of pathologies such as diabetes, cardiovascular disease and depression.

Moreover, this is further increased by reduced dietary choices, together with a high likelihood of overeating.

Some reviews support the notion that children with ASD could potentially benefit from lifestyle modifications that promote increased physical activity and sedentary behaviour. Participating in physical activity offers the possibility and enhancement of socialisation with peers, as well as opening up new interests and opportunities and increasing motor skills. This will have a positive impact on their quality of life including physical health, socio-emotional functioning and development [59], therefore, like typically developing (TD) children [20].

Programmes in which children were involved in controlled physical activity showed a great improvement in their quality of life and well-being, highlighting cognitive, communicational and motor aspects directly through physical exercise, as well as generating and improving the relationships of children and adolescents with their parents and family members [60]. Studies suggest that physical activity interventions for children with ASD should be implemented as early as possible to maximise physical activity while children are young and stem possible age-associated declines [20]. In this line, the recommendations resulting from our study, are in line with the needs detected in the aforementioned studies.

Along the article, we have described the key elements that professionals and supporting services must take into account in order to increase the participation of people with ASD in sports and physical activities. Some of them are coincident with the recommendations that authors such as Duquette et al. [61], propose in order to adapt supporting strategies to the communication needs of people with ASD.

Those same authors conclude that the activities, including those related to the sport practice, must consider and integrate the “individuality” of each person with ASD. To achieve this goal is necessary to know the person and his or her interests, as well as his or her needs and strengths. The system of quality of life indicators and specifically those related to physical wellbeing facilitate obtaining this information, even when the person with ASD can't communicate or provide it by itself.

In addition, these indicators can facilitate the role of the supporting staff or “sport practice coaches”. They must respond to the needs and skills of every person with ASD, sometimes without a specific training related to these kind of disorders. People on the autism spectrum need high levels of structuration on the schedules and activities, as well as specific supports to encourage their social participation. The sport trainers must know the person capacities and needs, and must adapt the sport practice responding to them. They should plan the activities in order to be clear and predictable to the person, and increase his or her comprehension and participation. However, they should be flexible and permit subsequent modifications in order to respond to the person needs [62]. The system of indicators can facilitate this planning and the resolution of unforeseen events, and can improve the practice of non-specialized in autism professionals.

Likewise, the results of this study increase the evidence about the perceptions and opinions that people with ASD express about their quality of life and the elements that improve it. Specifically, those results confirm that the sport practice can provide an opportunity for people with ASD to enjoy themselves, sharing it with peers and educators [63].

The results and the system of indicators contribute to guarantee and make real the fundamental right that people on the autism spectrum have to participate and be actively included in the society, as the *International Convention on the rights of persons with disabilities* establishes [2].

The contribution of the results and the guidelines related to them are significant for the educational field too. The empowerment in achieving personal goals and the improvement of the quality of life are first order objectives in the education of people with ASD. The implementation of a global framework and a system of quality of life indicators that guide the design of personal supporting plans in health educational programs is essential in order to assure a person-centered approach. In addition, these results contribute to the evaluation of programs effects, especially in relation to their impact on the real lives of people with ASD.

The results and recommendations obtained in the study are valuable resources to apply in training programs on ASD, especially in those oriented to train professionals [education, health, social services…]. The guidelines can help those professionals to know more about autism, but especially to improve their capacities to support and adapt their knowledge to the individual needs of each person with ASD.

Nevertheless, the study and the results present some limitations and improvement areas. First, all the participants [people on the autism spectrum, their families and professionals] receive services and supports from a network of specialized organizations. This is important because their perspectives and opinions must be different from those people with ASD who do not receive any support from specialized services.

Secondly, most participants with ASD had good communication skills and expressed their opinions by themselves and only a small group of participants presented ASD, intellectual disability and severe communication skills. There is an enormous difficulty in capturing the experiences and the perceptions of people with ASD and complex needs, often excluded from consultation processes about their lives [64, 65, 66].

Researchers and practitioners should improve the efforts to develop innovative methodologies that increase the participation of people with cognitive and communication difficulties in qualitative research. Quality of life is determined by both objective and subjective factors, so the consultation methodologies in research must combine both types of measures [67, 68, 69]. Increased collaboration between researchers and practitioners is essential to adapt the qualitative research techniques to the diversity presenting in the society, including people with cognitive issues and communication difficulties [like some people with ASD]. Developing inclusive research methodologies and transferring their results into applied resources is essential to improve the implementation of evidence-based practices and to produce positive impacts on the lives of those involved, including people with ASD and their families [70].

## 5 Conclusions

More information is needed in order to obtain a bigger picture of the needs, expectations and priorities of people with ASD and their families in relation to their quality of life. However, we think that the methodology and results of this project constitute a first approach in Spain to increase knowledge regarding the quality of life and the autism spectrum disorders. The project implies a first attempt to include people with ASD and severe supporting needs into inclusive models of qualitative research, which will be improve and developed in future studies.

It would be pending to deepen for future studies in some aspects such as the relationship between quality of life with other variables such as parents' educational level, age, degree of affectation in autism, age of people with ASD, relationship with other pathologies, etc., with a larger sample size in order to guarantee the representativeness of the study.

## Declarations

### Author contribution statement

Jose Luis Cuesta-Gomez; Ruth Vidriales-Fernández: Conceived and designed the experiments; Performed the experiments; Analyzed and interpreted the data; Contributed reagents, materials, analysis tools or data; Wrote the paper.

Raquel De la Fuente-Anuncibay; Maria Teresa Ortega Camarero: Conceived and designed the experiments; Analyzed and interpreted the data; Contributed reagents, materials, analysis tools or data; Wrote the paper.

### Funding statement

This research did not receive any specific grant from funding agencies in the public, commercial, or not-for-profit sectors.

### Data availability statement

Data included in article/supplementary material/referenced in article.

### Declaration of interests statement

The authors declare no conflict of interest.

### Additional information

No additional information is available for this paper.
